# N-glycoproteome Analysis of the Secretome of Human Metastatic Hepatocellular Carcinoma Cell Lines Combining Hydrazide Chemistry, HILIC Enrichment and Mass Spectrometry

**DOI:** 10.1371/journal.pone.0081921

**Published:** 2013-12-04

**Authors:** Xianyu Li, Jing Jiang, Xinyuan Zhao, Jifeng Wang, Huanhuan Han, Yan Zhao, Bo Peng, Rugang Zhong, Wantao Ying, Xiaohong Qian

**Affiliations:** 1 The College of Life Science and Bio-engineering, Beijing University of Technology, Beijing, China; 2 State Key Laboratory of Proteomics, Beijing Proteome Research Center, Beijing Institute of Radiation Medicine, Beijing, China; King's College London, United Kingdom

## Abstract

Cancer cell metastasis is a major cause of cancer death. Unfortunately, the underlying molecular mechanisms remain unknown, which results in the lack of efficient diagnosis, therapy and prevention approaches. Nevertheless, the dysregulation of the cancer cell secretome is known to play key roles in tumor transformation and progression. The majority of proteins in the secretome are secretory proteins and membrane-released proteins, and, mostly, the glycosylated proteins. Until recently, few studies have explored protein N-glycosylation changes in the secretome, although protein glycosylation has received increasing attention in the study of tumor development processes. Here, the N-glycoproteins in the secretome of two human hepatocellular carcinoma (HCC) cell lines with low (MHCC97L) or high (HCCLM3) metastatic potential were investigated with a in-depth characterization of the N-glycosites by combining two general glycopeptide enrichment approaches, hydrazide chemistry and zwitterionic hydrophilic interaction chromatography (zic-HILIC), with mass spectrometry analysis. A total of 1,213 unique N-glycosites from 611 N-glycoproteins were confidently identified. These N-glycoproteins were primarily localized to the extracellular space and plasma membrane, supporting the important role of N-glycosylation in the secretory pathway. Coupling label-free quantification with a hierarchical clustering strategy, we determined the differential regulation of several N-glycoproteins that are related to metastasis, among which AFP, DKK1, FN1, CD151 and TGFβ2 were up-regulated in HCCLM3 cells. The inclusion of the well-known metastasis-related proteins AFP and DKK1 in this list provides solid supports for our study. Further western blotting experiments detecting FN1 and FAT1 confirmed our discovery. The glycoproteome strategy in this study provides an effective means to explore potential cancer biomarkers.

## Introduction

Hepatocellular carcinoma (HCC) is a common malignant neoplasm and a major cause of cancer-related deaths in Asian countries. A high mortality rate for HCC is principally caused by uncontrolled tumor invasion and metastasis[1]. Cancer cell metastasis involves intricate, multi-step processes and various cytophysiological changes, including changes in the crosstalk between cells and the components in the extracellular space[2]. Over the past several years, the progress in the analysis of the human plasma proteome has provided a tremendous opportunity for discovering clinical biomarkers[3]. However, the complicated components of the plasma proteome and the wide dynamic range of concentration of these molecules present challenges for the discovery of new candidates[4].

Tumor cell secretory proteins could be specifically profiled without the depletion of high abundance serum proteins by culturing tumor cells in serum-free conditioned medium for a short duration, collecting the conditioned medium and subjecting it to proteome analysis. Over the past several years, a considerable amount of efforts has been focused on the analysis of cell secretomes to identify reliable and useful cancer biomarkers. In a representative study, the secretomes of a panel of cancer cell lines were generated, with the detection of 4,600 proteins from 23 cell lines[5]. High quality quantitative analysis of cancer cell secretomes has also been accomplished by combining azidohomoalanine labeling and stable isotope labeling with amino acids in cell culture[6].

Protein glycosylation has been directly linked with cancer development[7]. Almost all of the currently used protein biomarkers are secreted glycoproteins such as carcinoembryonic antigen (CEA), cancer antigen 125 (CA125), prostate specific antigen (PSA) and alpha-fetoprotein (AFP)[8]. Because most of the proteins in the secretory system are glycosylated[9], it is natural to expect that the glycoproteomic analysis of the tumor cell secretome will provide valuable biomarkers. While many studies have been performed for the in-depth profiling of glycoproteins in the plasma, efforts to profile the glycoproteins of secretory proteins from the conditioned medium (CM) are still rather preliminary[10]. Only limited research has been performed to explore the N-glycosylation changes of the secretome that are derived from hepatocellular carcinoma cells, despite great biological and clinical interests[11]. With the recent advances in proteomic technologies, liquid chromatography-mass spectrometry (LC-MS) has become the key tool for analyzing post-translational modifications (PTMs) such as phosphorylation, ubiquitination, acetylation and N-glycosylation[12]. Similar to other PTMs, specific enrichment is essential to capture the often low abundance glycopeptides. Several enrichment methods, including lectin affinity[13,14], hydrophilic interaction[15] and solid phase extraction using hydrazide chemistry[16], have been developed and applied for characterizing N-glycoproteins and N-glycosites[17].

Here, we set out to profile the N-glycoproteins that are secreted by HCC cell lines with low (MHCC97L) or high (HCCLM3) metastatic potential. The two cell lines are derived from the same genetic background[18]. For improved coverage, hydrazide chemistry[19] and zic-HILIC[15] were tested for the enrichment of N-glycopeptides. After duplicate biological analyses, a total of 1,213 unique N-glycosites from 611 N-glycoproteins from both cell lines were confidently identified. A label-free approach was used to quantify the differences in the signal intensity of the MS response between the metastatic cell lines[20,21]. Our study provided clues for the involvement of a few glycoproteins in the metastasis processes, and the differentially regulated proteins may result in the discovery of novel candidates for the measurement of metastasis.

## Results

### Analytical strategy

**Figure 1:**
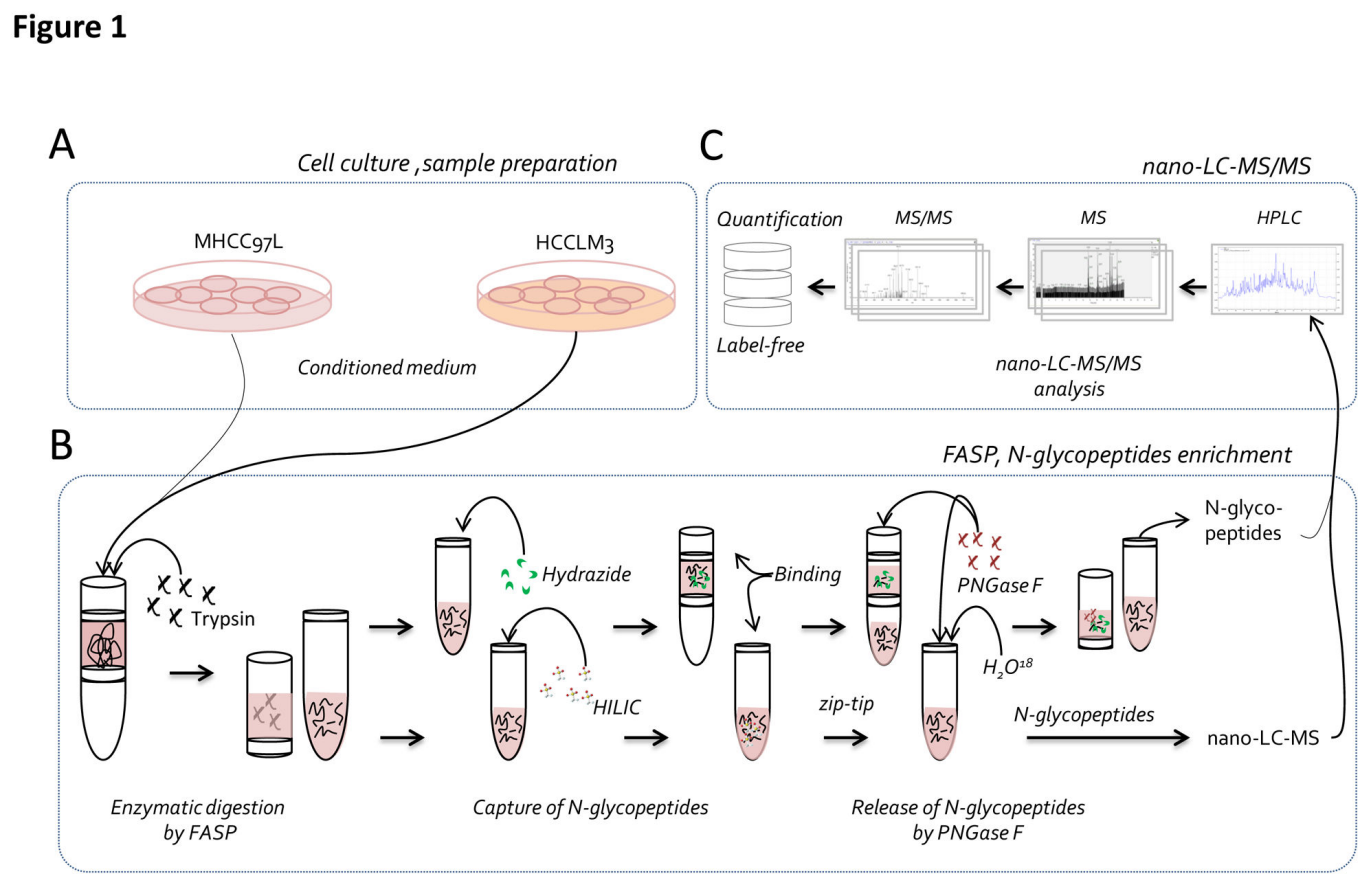
Overview of the experimental workflow. A) The secretome was collected from the conditioned medium. B) N-glycosylated peptides were enriched using hydrazide chemistry and zic-HILIC methods. First, proteins were digested using FASP, and then the N-glycosylated peptides were captured using two methods, followed by de-glycosylation using PNGase F and LC-MS-MS analysis. C) Label-free quantitative analysis.

Figure 1 is a schematic representation of our experimental approach. To investigate the proteins that are related to liver cancer metastasis, we followed this workflow to profile the difference of N-glycosylated proteins in the secretome of HCC cell lines with different metastatic potential. To reduce the interference from high abundant serum proteins, the culture medium was not supplemented with serum[10,22,23]. An initial experiment showed that none of the metastatic cell lines suffered from significantly reduced viability after 24 h of culturing in the CM. Thus, a culture time of 24 h and the DMEM CM without serum were selected as the essential conditions for culturing the cells[18]. Figure 1A shows that the procedures for the collection of the secretomes. The CM was centrifuged and precipitated with TCA. Protein digestion was performed using the filter aided sample preparation (FASP) method. After the digestion, N-glycopeptides were captured by hydrazide chemistry and HILIC methods (Figure 1B). 

Next, the enriched N-glycopeptides were processed by PNGase F to remove the glycans. The reaction was conducted in H_2_^16^O for the hydrazide method and in H_2_^18^O for the HILIC method. For the former, the peptide masses showed a 0.9848 Da increase due to the conversion of asparagine to aspartic acid, and for the latter, a 2.9883 Da increment was detected because of the introduction of an ^18^O atom during the PNGase F cleavage[10,17,24]. The deglycosylated peptides were detected using LC-ESI-MS/MS. The LC-ESI-MS/MS data were searched against a human proteome database using Proteome Discoverer software. The matched precursor ion area information was further used for label-free quantification (Figure 1C). 

### Mapping N-glycopeptides in the secretomes

**Figure 2:**
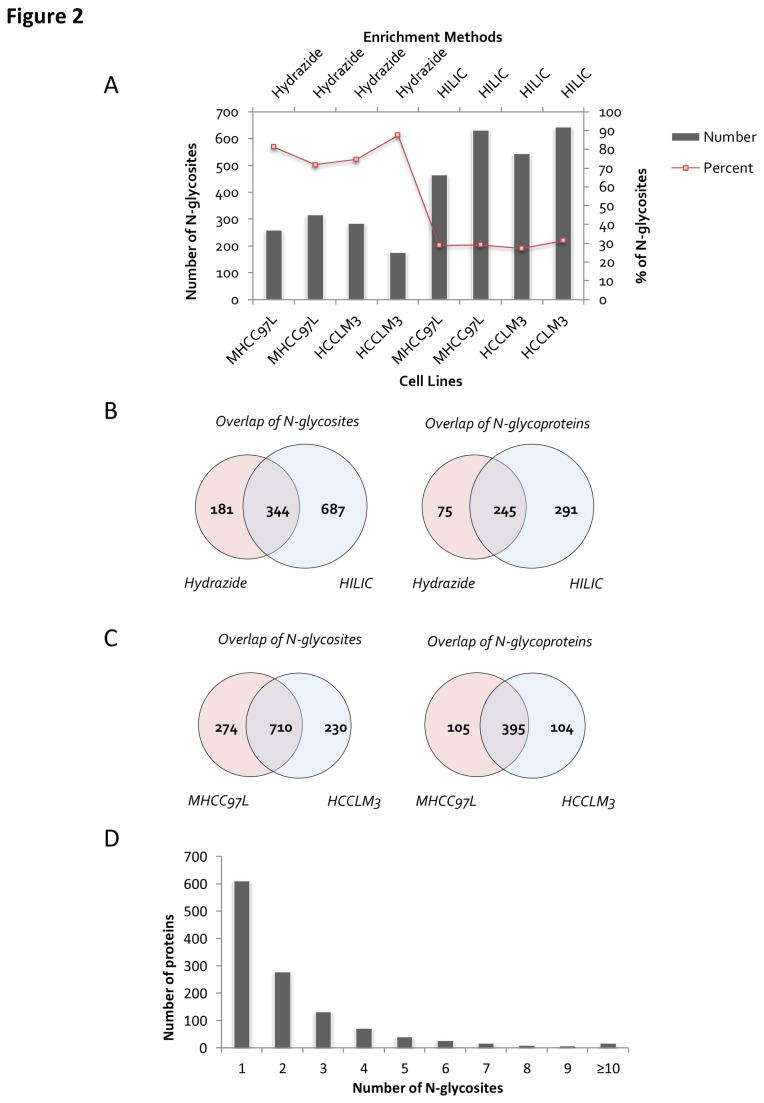
Summary of identified N-glycosites. A) The number of unique N-glycosites identified and the percentage of N-glycopeptides from all of the identified peptides in each cell line. B) Overlap of N-glycosites between the different enrichment methods. C) Overlap of the N-glycosites and proteins between the different cell lines. D) Number of N-glycosites identified per protein.

A total of 100 μg FASP digested peptides (corresponding to 200 μg initial proteins) was used for each experiment with ~100 μL hydrazide-modified agarose beads or 2 mg zic-HILIC material used for the enrichment. Two biological replicates were conducted. The details of the protein identifications are described in Table S1. Figure 2A shows the enrichment specificity, which could be estimated based on the percentage of N-glycopeptides among all of the identified peptides, and the enrichment efficiency, which could be determined based on the number of identified unique N-glycosites. Although the hydrazide chemistry method showed higher specificity than zic-HILIC for the identification of glycopeptides (80 % vs. 30 %, as indicated by the trendline in Figure 2A), the former provided a much lower number of identified N-glycosites than the latter (280 vs. 600 on average). Compared with other studies using similar enrichment methods[10,17,21], higher numbers of N-glycosites and glycoproteins were identified here (526 unique N-glycosites and 320 unique glycoproteins from hydrazide chemistry, and 1,032 unique N-glycosites and 536 unique glycoproteins from the zic-HILIC method), of which 344 N-glycosites and 245 unique glycoproteins were identified by both methods (Figure 2B); the other N-glycosites and glycoproteins were obtained from hydrazide or zic-HILIC alone (181 vs. 687 N-glycosites and 75 vs. 291 glycoproteins, as shown in Figure 2B). Therefore, the two enrichment methods were somewhat complementary.

Figure 2C shows the overlap of unique N-glycosites and unique N-glycoproteins between the MHCC97H and HCCLM3 cell lines. Based on the two enrichment methods, 948 and 907 unique N-glycopeptides were detected from MHCC97L and HCCLM3 cell lines, respectively, and the degree of overlap for the N-glycosites (984 vs. 940) and N-glycoproteins (500 vs. 499) that were identified from the two metastatic cell lines was ~60 % and ~66 %, which suggests a relatively high degree of similarity between the two cell lines.

A total of 1,165 unique N-glycopeptides and 1,213 N-glycosites were recognized and mapped to 611 glycoproteins (Table S2). Approximately 50 % of the glycoproteins were identified with one N-glycosite, nearly 34 % with two or three N-glycosites, and approximately 14 % with four to nine N-glycosites. The protein with the most N-glycosites was low-density lipoprotein receptor-related protein 1 (LRP1), with 18 identified N-glycosites (Figure 2D).

### Biological categorization of identified glycoproteins

**Figure 3:**
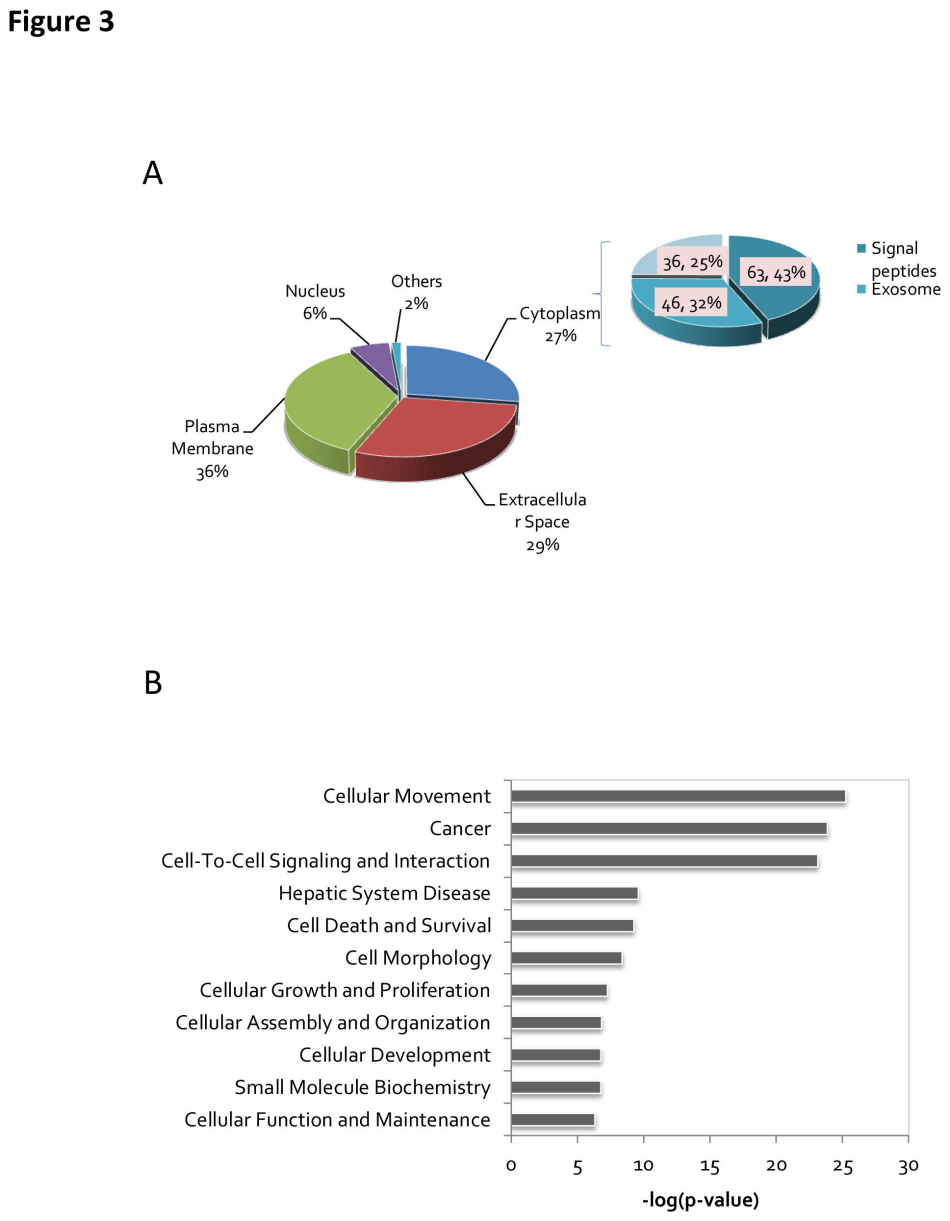
Bioinformatics analysis of identified N-glycoproteins. A) Cellular component annotation of identified N-glycoproteins. B) Biological functions of differentially expressed N-glycoproteins.

To determine what subset of proteins was enriched in the secretome, we performed a prediction analysis of the protein cellular localization using Gene Ontology (GO). Approximately 65 % of the glycoproteins identified were categorized into the extracellular space and plasma membrane in the GO subcellular localization annotation (Figure 3A) (Table S3). Although 27 % of the glycoproteins have a GO subcellular localization term of cytoplasm, 75 % of these proteins were predicted to contain signal peptides. Sixty-four of these cytoplasm-localized proteins contained signal peptides, as determined by Signal P 4.0, and forty-six of them were predicted to be localized to the exosome by comparing the data against an exosome database[25]. Taken together, 90% of the identified proteins were predicted to be secreted. Moreover, the enrichment scores for the clusters of glycoproteins and secretory proteins were 195.2 and 50.2, respectively, as compared to the human proteome background by DAVID analysis (*p*-values: 2.52×10^-204^ and 2.42×10^-63^; Benjamini: 1.26×10^-201^ and 3.03×10^-61^) (Table S4).

The most significantly enriched biological functions are listed in Figure 3B. The top 10 functional categories, according to their *p*-values, that are enriched in the metastatic cell lines are shown in Figure 3B and Table S5. It can be observed that the top category contains proteins related to cell movement function, which could be involved in cancer cell migration and invasion.

### Label-free quantitative analysis of N-glycopeptides

**Figure 4:**
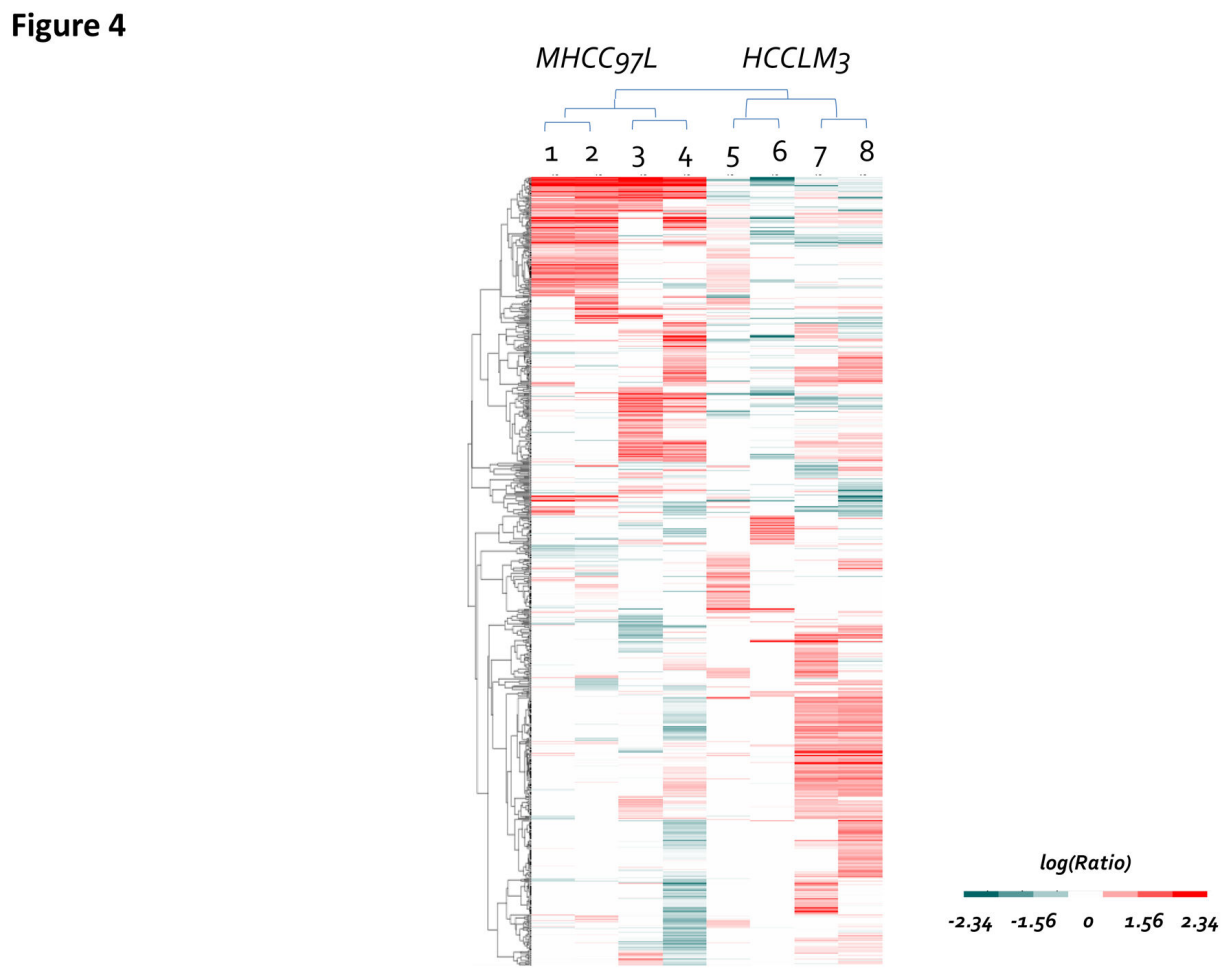
Label-free quantitative analysis. Hierarchical clustering based on the Euclidean distance of the peak area of label-free quantification of N-glycosites showed a significant difference between the MHCC97L and HCCLM3 cell lines.

To explore the differentially expressed glycoproteins, label-free quantitative analysis was performed. Peptide quantities were estimated by precursor ions areas, which were shown in Table **S1**. The differentially expressed glycopeptides between the two cell lines are shown in Table S6, of which 1,096 N-glycopeptides had quantification values. To avoid the possible interference from noise, the glycopeptides were normalized by the total precursor area of the identified peptides[20,21]. The quantitative information of the 683 overlapped N-glycopeptides between the two cell lines was filtered by hierarchical clustering (Figure 4) and segregated into 10 distinct clusters, including up-regulated, down-regulated or unchanged glycopeptides (Table 1). Clusters a) and b) contain the glycopeptides with higher relative abundance levels in the MHCC97L cell line, which includes 65 and 86 glycopeptides from the two enrichment methods. Clusters i) and j) contain the glycopeptides that have higher relative abundance in HCCLM3 cells, which includes 101 and 64 glycopeptides from the two enrichment methods (Table S6). Clusters c) - h) include more than 50% of the identified glycopeptides without any significant changes in abundance between the two metastatic cell lines.

**Table 1:** Ten major clusters extracted from Figure 4 by K-Means expression clustering.

**Description**	**Cluster**	**Number**	**Tendency**
The glycopeptides with higher relative abundance levels in the MHCC97L cell line	a)	65	MHCC97L >> HCCLM3
	b)	86	MHCC97L > HCCLM3
More than 50% of the identified glycopeptides without any significant changes in abundance between the two metastatic cell lines.	c)	62	No significant changes
	d)	51	
	e)	26	
	f)	83	
	g)	54	
	h)	86	
The glycopeptides have higher relative abundance in HCCLM3 cells	i)	101	MHCC97L < HCCLM3
	j)	64	MHCC97L << HCCLM3

In addition to the N-glycopeptides that were identified in both metastatic cell lines, there were 258 N-glycopeptides that were only observed in the MHCC97L cell line and 217 N-glycopeptides that were only observed in the HCCLM3 cell line. These glycopeptides belong to 385 unique N-glycoproteins. Among them, 84 % were identified with only one N-glycopeptide, while approximately 4 % of them were identified with 3 N-glycosites. Fibronectin 1 (FN1) was identified by 5 and 9 N-glycopeptides from the MHCC97L and HCCLM3 cell lines, respectively, and the quantitative analysis indicated that the relative abundance level of the former was far less than that of the latter. The 4 glycopeptides that were not identified in the MHCC97L cell line may have been of low abundance, or they could have been affected by altered FN1 glycosylation patterns under different metastatic potentials. From a biomarker perspective, the proteins that belong to clusters i) - j) in Table 1 and those that are uniquely expressed in the HCCLM3 cell line would be the most valuable, as these were up-regulated or detected in concordance with the increased HCC metastatic potential. Within these clusters, well-known hepatocellular carcinoma biomarkers such as alpha fetoprotein (AFP) and Dickkopf-related protein 1 (DKK1)[26] were identified, as well as proteins that have been suggested to be related to metastasis in other studies, for example, integrin alpha-3 (ITGA3) and tissue inhibitor of metalloproteinase-1 (TIMP-1)[21]. These glycoproteins are taken as the positive controls for our strategy, and the results indicate that the N-glycosylated secretome of the metastatic cell lines provides a potential source of disease markers.

### The network of metastasis-related glycoproteins

**Figure 5:**
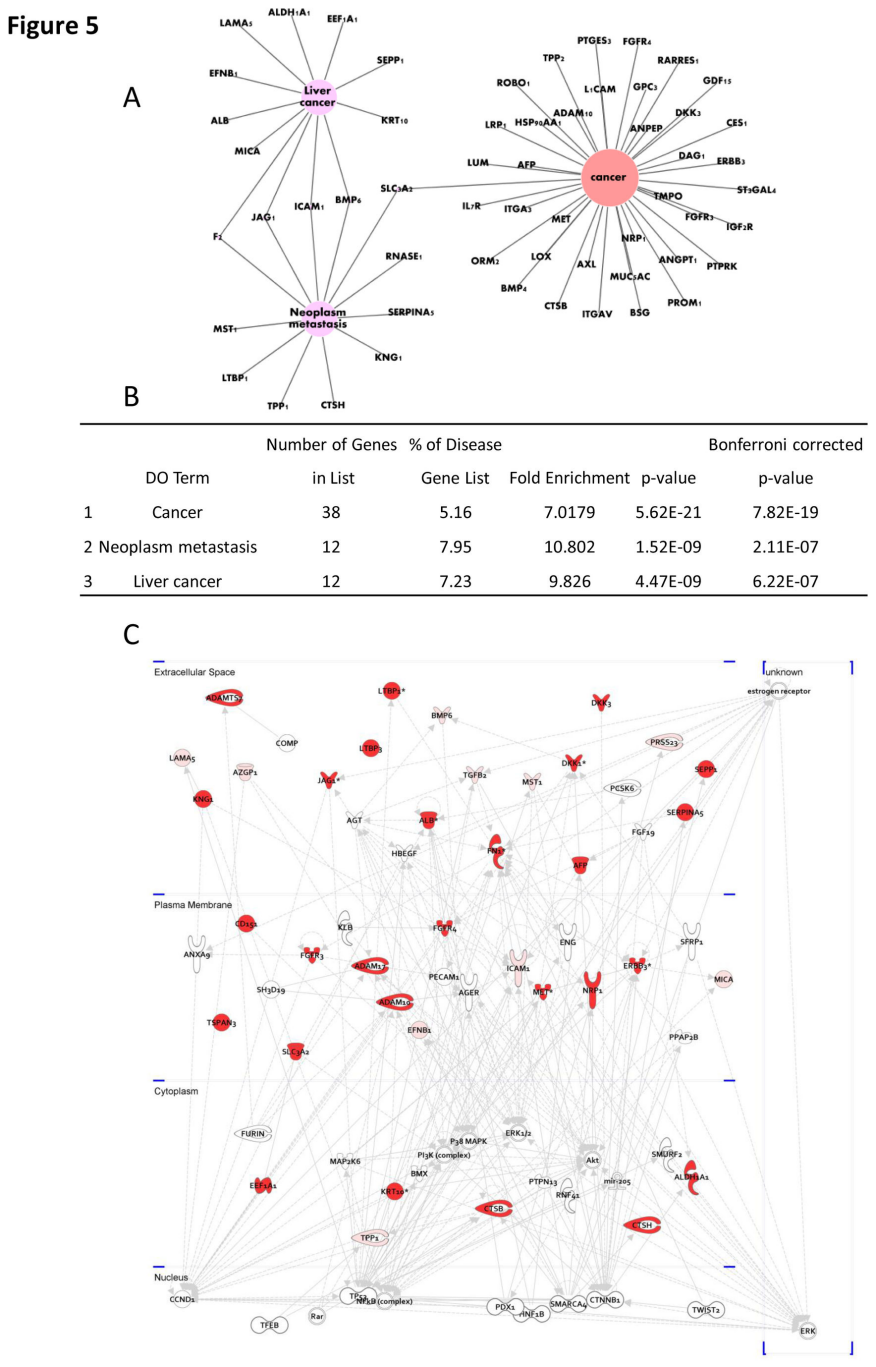
Network view of the up-regulated N-glycoproteins in HCCLM3 cells. A) The networks of the top 3 liver-related diseases. B) The number of related genes and the *p*-value of the top 3 liver-related diseases that are indicated in A). C) Cellular motility network. (The proteins with higher expression in HCCLM3 cells are in red (Ratio > 2), whereas the other proteins that were generated from the IPA database are not colored.).

To further elucidate the correlation of the identified N-glycoproteins with metastasis and other diseases, we assigned the glycoproteins containing up-regulated N-glycosites in the HCCLM3 cell line to different diseases using the web tool FunDO[27]. As shown in Figure 5A-B, approximately half of the 300 glycoproteins were related to cancer, liver cancer, and neoplasm metastasis (Bonferroni corrected *p*-values: 7.82×10^-19^, 6.22×10^-7^ and 2.11×10^-7^). To explore the key glycoproteins and substrates that are related to HCC metastasis, a metastatic network was constructed using the differentially expressed glycoproteins (Figure 5C). The significantly regulated liver cancer-related glycoproteins that were identified by FunDo and IPA are listed in Table 2. Their gene ontology (GO) information, including cellular component and biological processes, is also shown in Table 2.

**Table 2:** List of significantly up-regulated N-glycosites related to liver cancer in FunDo and IPA.

Protein Accessions	Peptide Sequence	Protein Name	Gene symbol	Expression level*	Quantitative ratio*	Extracellular location	Type
40548389	ASSEVNLANLPPSYHn*ETNTDTK	Dickkopf-related protein 3	DKK3	M >> L	∞	√	Cytokine
4502337	DIVEYYn*DSn*GSHVLQGR	Alpha-2-glycoprotein 1, zinc-binding	AZGP1	M > L	2.052	√	Transporter
47132557	DQCIVDDITYNVn*DTFHKR	Fibronectin 1	FN1	M > L	2.004	√	Enzyme
18497288	DSCQQGSn*MTLIGENGHSTDTLTGSGFR	Latent transforming growth factor beta binding protein 3	LTBP3	M >> L	∞	√	Other
261337165	ECYYNLNDASLCDNVLAPn*VTK	Latent transforming growth factor beta binding protein 1	LTBP1	M >> L	∞	√	Other
62530391	EGYSn*ISYIVVNHQGISSR	Selenoprotein P, plasma, 1	SEPP1	M >> L	∞	√	Other
4502425	Fn*LSQIPEGEVVTAAEFR	Bone morphogenetic protein 6	BMP6	M > L	2.024	√	Growth factor
38683827	Fn*LTANQHLLAPGFVSETR	ADAM metallopeptidase with thrombospondin type 1 motif, 7	ADAMTS7	M >> L	∞	√	Peptidase
21265058	GAEYVISPLPn*ASAPAAQR	ADAM metallopeptidase with thrombospondin type 1 motif, 15	ADAMTS15	M >> L	∞	√	Peptidase
47132557	GGNSNGALCHFPFLYNNHn*YTDCTSEGR	Fibronectin 1	FN1	M >> L	64.92	√	Enzyme
4501989	Vn*FTEIQK	Alpha-fetoprotein	AFP	M >> L	∞	√	Transporter
205277383	GTAn*TTTAGVPCQR	Hepatocyte growth factor-like	MST1	M > L	4.103	√	Growth factor
7110719	IQKDHHQASn*SSR	Dickkopf-related protein 1	DKK1	M >> L	∞	√	Growth factor
47132557	ISCTIAn*R	Fibronectin 1	FN1	M >> L	∞	√	Enzyme
4507463	LTSPPEDYPEPEEVPPEVISIYn*STR	Transforming growth factor-beta 2	TGFB2	M >L	4.429	√	Growth factor
4557679	n*CSHLKDHCR	Jagged 1	JAG1	M >> L	∞	√	Growth factor
6005882	QYLSYETLYAn*GSR	Protease, serine, 23	PRSS23	M >L	8.103	√	Peptidase
4506547	Rn*MTQGR	Ribonuclease, RNase A family, 1	RNASE1	M >> L	∞	√	Enzyme
4505821	TFYWDFYTn*R	Prolactin-induced protein	PIP	M >> L	∞	√	Other
21264602	TLSELMSQTGHLGLAn*ASAPSGEQLLR	Laminin, alpha 5	LAMA5	M > L	2.616	√	Other
194018472	VLPSLGISNVFTSHADLSGISn*HSNIQVSEMVHK	Serpin peptidase inhibitor, clade A member 5	SERPINA5	M >> L	∞	√	Other
262050546	YNSQn*QSNNQFVLYR	Kininogen 1	KNG1	M >> L	∞	√	Other

L: MHCC97L and M: HCCLM3. *: >/ >> The expression level of N-glycopeptides in HCCLM3 was more than two/ ten times higher than that in the MHCC97L cell line; >>>: only expressed in the HCCLM3 cell line.

### Validation of up-regulated glycoproteins

**Figure 6:**
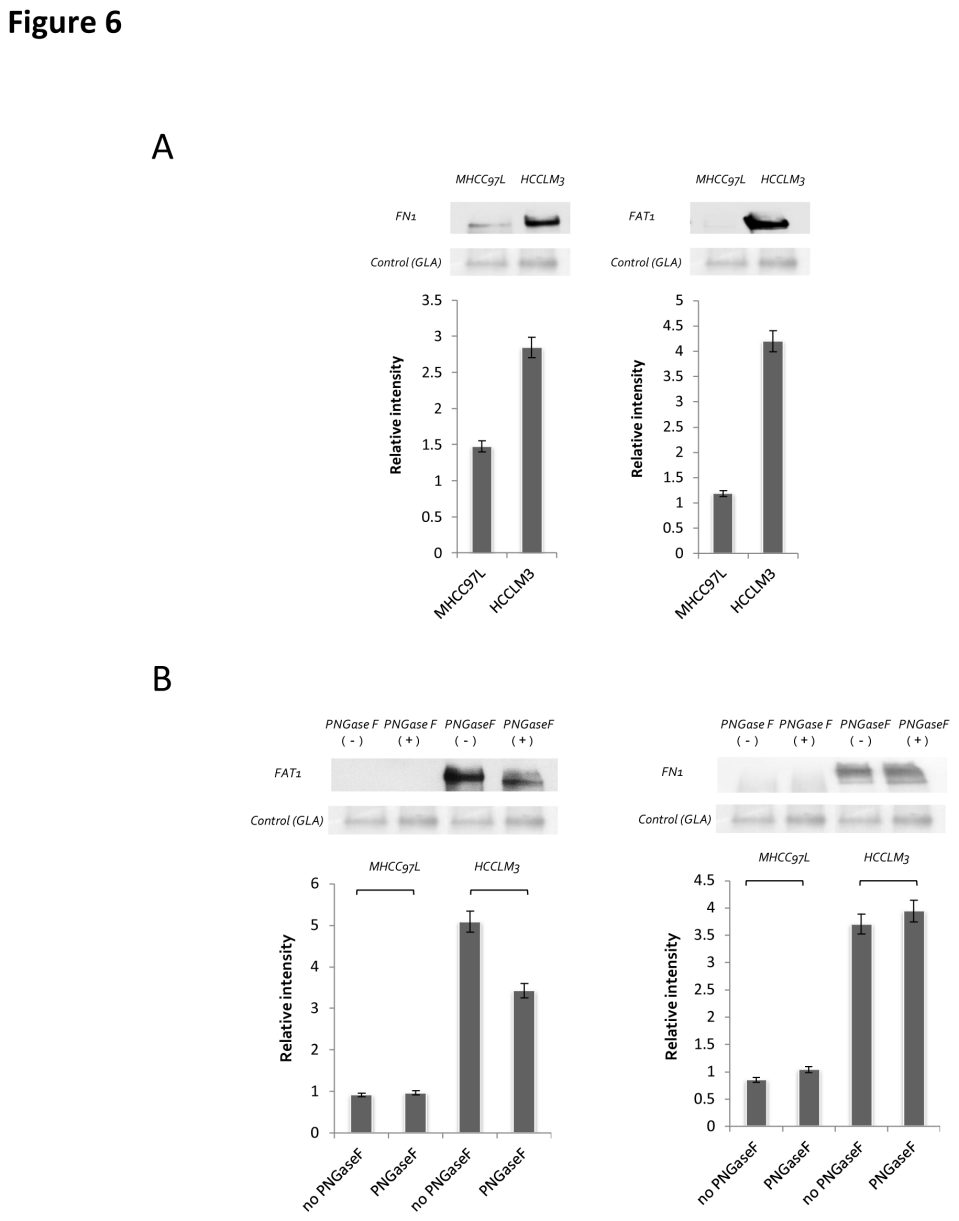
Validation of the differential expression of two selected N-glycoproteins. A) Ten micrograms secretome protein samples were separated on SDS-PAGE gels, transferred to PVDF membranes, and probed with anti-FN1 or FAT1 antibodies. B) The de-glycosylation of the same amount of secreted proteins from MHCC97L and HCCLM3 cells was performed with PNGase F cleavage for 12 h. Proteins were separated on SDS-PAGE and analyzed by western blotting.

Among these glycoproteins containing up-regulated N-glycosites, FN1 and FAT1 were selected for further validation via western blotting analysis (Figure 6A). The protein alpha-galactosidase A (GLA) was selected as a loading control because it is expressed equally in the cell lines[28]; its ratio was also close to 1 based on the mass spectrometry (MS) data (Table S6) and the western blot results (Figure 6A). To measure the glycosylated status of the two proteins, the same experiment was repeated with the addition of PNGase F processing (Figure 6B). The results showed that for FAT1, an apparent shift to the low molecular weight region was observed. While for FN1, the shift was not significant. This could be caused by the huge molecular weight of FN1 (260 KDa), thus, the releasing of glycans from the protein sequence did not induce a discriminable shift. 

## Discussion

Our work represents an in depth glycoproteomics analysis of the secretome of HCC cell lines with different metastatic potentials. By applying hydrazide chemistry and zic-HILIC for glycopeptide enrichment, followed by high mass accuracy LC-MS/MS analysis, N-glycopeptides and specific N-glycosites were identified with high confidence[17,24,28]. Our results demonstrate that the agarose bead-based hydrazide chemistry offers a highly specific means of enriching N-glycosites from the secretome, and the specificity was higher than 80 %[17]. Although many non-glycopeptides were identified, the zic-HILIC enrichment method proved to be a more effective strategy because approximately 600 N-glycosites were identified per experiment. The combination of both methods could significantly improve the identification of N-glycosites.

The expression of the glycoproteins in the secretory system reflected the intracellular regulation of the N-glycosite synthesis, transport and secretory pathway, which could be illustrated through IPA and DAVID analysis[29]. Among these biological functions, the most significant class, cell movement, related to cancer cell migration and invasion was observed, in which, cathepsin B (CTSB), fibronectin (FN1), CD44 antigen (CD44), extracellular matrix protein 1 (ECM1), and protocadherin Fat 1 precursor (FAT1) previously shown to play an important role during the metastasis of other cancer cells[30] were included.

Four hundred forty-seven N-glycosites from 300 glycoproteins were found to be significantly altered with a ratio > 2 (significantly different with *p* < 0.05, as determined by SPSS analysis), including clusters i) and j) in the label-free quantification and the non-overlapping dataset from HCCLM3. Validation by western blot analysis exhibited the same trends as the mass spectrometry intensity (Figure 6), which further supports the observation that most of the glycoproteins could play a role in metastasis. Next, we focused on the function analysis of these up-regulated glycoproteins because they represent potential diagnostic maker candidates for predicting hepatocellular carcinoma metastasis and because they are drug targets for inhibiting metastasis.

The growth factor receptors ErbBs and FGFRs have been reported to be associated with breast cancer development. Two and three of their N-glycosites were identified in this study, respectively[31]. PRSSs (one identified glycosite) and latent TGF-beta binding proteins (LTBPs, five identified glycosites) are involved in growth factor signaling and were included in the detection list. CD151, identified with one N-glycosylation site, is a cell surface glycoprotein that is known to enhance the motility, invasion and metastasis of cancer cells[32]. MET, reported as a hepatocyte metastasis-related protein, is a membrane receptor that is essential for embryonic development and wound healing [33]. Seven N-glycosites of MET proteins were identified in our dataset, and three had a higher relative abundance in the HCCLM3 secretome. 

Although the aforementioned proteins are involved in tumor invasion and metastasis, they could have low specificity for hepatocellular carcinoma. Combining the prediction results by FunDO[27] that produced a list of liver cancer and metastasis related proteins with IPA network analysis, we obtained 20 glycoproteins that could be related to liver cancer metastasis (Table 2). All of these proteins are localized to the extracellular space. For example, hepatocyte growth factor-like protein in humans is encoded by the MST1 gene that is involved in cell apoptosis and metabolism, and it exhibited a higher relative abundance in the high metastatic cell line[34]. BMP6 is the key regulator of hepcidin, a small peptide secreted by the liver and acted as a major regulator of iron metabolism in mammals[35]. In the IPA network (Figure 5C), the molecule that is upstream of BMP6 is DKK1, and both of them were highly expressed at the N-glycosylation level. Because DKK1 has been reported as an important marker of hepatocellular carcinoma[26], we reasoned that this pathway could be significantly involved in liver cancer metastasis and invasion.

Synergistic secretory proteins were also among the up-regulated proteins in this study. For example, zinc alpha2 glycoprotein (AZGP1) is expressed in epithelial secretory cells[36] and forms a complex with PIP; their co-up-regulation has been detected in well-differentiated tumors[37]. TGF-beta is regulated by its association with LTBPs [38,39]. LTBP1, LTBP3 and TGFβ2 were all up-regulated in the HCCLM3 cell line. ADAMTS[40], selenoprotein (SEPP1)[41], ICAM1[42], JAG1[43], and LAMA5[44] have been demonstrated to have important roles in connective tissue organization, angiogenesis and cell migration. The label-free quantification results implied that they could play an important role in hepatocellular carcinoma metastasis.

Additionally, we found that FN1 had a higher level of expression in HCCLM3 cells (9 N-glycosites) and a lower level in MHCC97L cells (5 N-glycosites), which was further verified by western blot (Figure 6A). FAT1 was only detected in the HCCLM3 cell line (2 N-glycosites), and its expression was also verified by western blot (Figure 6A). The removal of glycans by PNGase F affected the migration of the protein in the gel, indicating its fully glycosylation (Figure 6B). 

## Conclusion

In summary, we used a glycoproteomics approach to investigate the secretome of hepatocellular carcinoma cell lines with different metastatic potentials. The qualitative and quantitative analysis of N-glycopeptides from the secretomes unveiled a panel of glycoproteins that may be related to hepatocellular carcinoma metastasis. It should be noted that our study focused on the difference of N-glycosites, and the regulation on the intact proteins were not examined, which mean that some of the differences at the N-glycosites level may be resulted from the differential expression of intact proteins. Dedicated experiments should be designed to consider both the changes at the protein level and glycosylation level. Nevertheless, our results still proved that it is efficient to find potential biomarkers through the capture of glycopeptides and its quantitative application, which was supported by the discovery of several well-known markers that were partially validated in our study. The glycoproteomics approaches we used, and the differentially expressed proteins that we identified, could benefit further research that seeks to discover early diagnostic or therapeutic biomarkers for hepatocellular carcinoma metastasis, and it could also provide a general strategy for other disease-related secretome studies. 

## Materials and Methods

### Cell culture and sample preparation

The human metastatic HCC cell lines MHCC97L and HCCLM3 were obtained from the Liver Cancer Institute of Zhongshan Hospital, which is affiliated with Fudan University (Shanghai, China). Their genetic backgrounds are similar because they are filial-cloned sequentially from a MHCC97 parental cell line[18,45]. The metastatic potential of the HCCLM3 cell line is higher than that of the MHCC97L cell line[46]. After orthotopic implantation of HCCLM3 tumor tissue into nude mouse liver for 35 days, widespread loco-regional and distant metastases were found 100% in lungs and abdominal wall, 80% in intra-abdominal cavity, and 70% in diaphragm[45,47]. While the pulmonary metastatic rate of MHCC97L was only 40 % [48]. Immunocytochemical studies demonstrated that the two clones were positive for AFP and the concentration of serum AFP was higher in HCCLM3-inoculated mice than that of MHCC97L[49]. The cell lines with different metastatic potential provides an important model system for the in vivo and in vitro study of HCC metastasis[50].

Approximately 1×10^7^ cells were cultured at 37 °C in 5 % CO_2_ in DMEM (Hyclone, USA) supplemented with 10 % fetal bovine serum until reaching 60 ~ 70 % confluence. Cells were washed stringently and gently four times with Dulbecco’s phosphate buffered saline with calcium and magnesium (DPBS) and once with serum-free DMEM (Conditioned Medium, CM). Cells were then incubated in the CM at 37 °C for 24 h. The secretory proteins were collected and centrifuged at 2,500 ×g for 10 min (4 °C) to remove the detached cells and large debris. The resulting supernatant was collected and centrifuged for 1 h at 100,000 ×g (4 °C) to remove smaller debris and vesicles. Formic acid (FA, final concentration of 0.1 %) was immediately added to the final supernatant, which was then stored at -80 °C. The addition of FA lowered the pH (pH < 4) of the culture supernatants, thus reducing the activity of many proteases[11,22].

The proteins in the culture supernatants were extracted by trichloroacetic acid (TCA) precipitation. Thirty milliliters of culture supernatants were used for each precipitation. All of the treatments were performed at 4 °C. TCA was added to the CM solution to a final concentration of 12 %[23]. After mixing, the solution was precipitated for 4 h at 4 °C, followed by centrifugation (10,000 ×g, 10 min, 4 °C). The protein pellet was carefully washed twice with 2 mL tetrahydrofuran (THF) (pre-cooled on ice), and each wash was followed by centrifugation (10,000 ×g, 10 min, 4 °C). The final pellet was re-dissolved in 0.4 mL lysis buffer (4 % SDS, 0.1 M Tris-HCl, pH 7.6, 100 mM DTT) with a sonicator bath (30 min extraction). The concentrations of the extracted proteins were measured by a NanoDrop spectrophotometer (Thermo, USA) at 280 nm with an extinction coefficient of 1.1 absorbance units[51].

### Protein digestion by FASP

Detergent was removed from the lysates, and the proteins were digested with trypsin using the FASP protocol[51], which entails the ultrafiltration of spin units with a nominal molecular weight cut-off of 30,000. Briefly, to YM-30 Microcon filter units (Millipore, USA) containing the CM protein concentrates, 200 μL 8 M urea in 0.1 M Tris/HCl, pH 8.5 (UA buffer) was added, and the units were centrifuged at 14,000 ×g at 20 °C for 15 min. This step was repeated twice. Then, 50 μL 0.05 M iodoacetamide in 8 M urea was added to the filters and the samples were incubated in the dark for 20 min. The filters were washed twice with 100 μL 8 M UA buffer followed by three washes with 100 μL 50 mM NH_4_HCO_3_. Finally, trypsin (Promega, USA) was added in 100 μL 50 mM NH_4_HCO_3_ to each filter. The protein to enzyme ratio was 100:1. The samples were incubated overnight at 37 °C, and the digested peptides were collected by centrifugation at 14,000 ×g at 20 °C for 15 min.

### N-glycopeptide enrichment by hydrazide

N-glycopeptides were captured from tryptic peptide mixtures by hydrazide chemistry following the protocol of Zhang[19] with modifications. Briefly, an aliquot of 100 μg peptides was diluted with coupling buffer (50 % acetonitrile, 0.5 % acetic acid) to a final reaction environment and then oxidized with 20 mM sodium periodate (NaIO_4_) at room temperature in the dark for 1 h. The excess NaIO_4_ was removed after oxidation by desalting using a Sep-Pak C18 column (Waters, USA). The oxidized peptides in 800 μL coupling buffer (80 % acetonitrile, 0.5 % acetic acid) were then incubated overnight at room temperature with hydrazide beads (100 μL beads for ≤ 100 μg peptides) that were pre-washed twice with 1 mL water. Unbound peptides in the supernatant were removed by centrifugation. The beads were then washed 3 times with 2 mL water, 3 times with 2 mL 1.5 M NaCl and 3 times with 50 mM NH_4_HCO_3_ to remove the non-glycopeptides. The captured N-glycopeptides were released by incubating the beads with 3 μL PNGase F (500 units/μl, New England Biolabs, USA) in 100 μL 50 mM NH_4_HCO_3_ buffer (dissolved in ^18^O water) at 37 °C for 12 h. The released glycopeptides were collected from the supernatant, and the hydrazide beads were washed twice with 200 μL 80 % ACN and 0.1 % FA. The washing solutions were combined with the supernatant collected from the PNGase F incubation and dried down to a final volume of 20 μL using a SpeedVac concentrator.

### N-glycopeptide enrichment by HILIC

The digested peptides were enriched with zic-HILIC media by the procedure reported by Calvano[15] with slight modifications. Briefly, first, the C8 disk was inserted into a 200 μL tip. Approximately 100 μg in-solution digested samples was dissolved in 80 % ACN, 0.5 % FA and incubated overnight at room temperature with 2 mg zic-HILIC media (Merck; particle size 10 μm) that was pre-washed twice with 1 mL coupling buffer (80 % ACN, 0.5 % FA). Then, zic-HILIC media was loaded into the 200 μL tip that was pre-filled with a C8 disk. The zic-HILIC tip was washed 5 times with 100 μL 80 % ACN, 1 % FA, 19 % H_2_O, and the bound peptides were eluted 3 times with 80 μL elution buffer (99 % H_2_O, 1 % FA). The eluate was dried, de-glycosylated with 3 μL PNGase F (500 units/μL, New England Biolabs) in 50 mM NH_4_HCO_3_ (dissolved in ^18^O water) at 37 °C overnight and dried down to a final volume of 20 μL using a SpeedVac concentrator.

### LC MS/MS analysis

A nanoflow HPLC instrument (EASY-nLC 1000 system, Thermo Fisher Scientific, USA) was coupled on-line to a Q-Exactive mass spectrometer (Thermo Fisher Scientific, USA) with a nanoelectrospray ion source (Thermo Fisher Scientific, USA)[10]. Chromatography columns were packed in-house with Ultimate XB-C18 3 μm resin (Welch Materials, USA). The peptide mixtures were loaded onto the C_18_-reversed phase column (10 cm length, 75 μm inner diameter) with buffer A (99.5 % water and 0.5 % FA) and separated with a 75 min linear gradient of 3 - 100 % buffer B (99.5 % acetonitrile and 0.5 % FA) at a flow rate of 350 nL/min. Including the loading and washing steps, the total time for an LC MS/MS run was approximately 90 min. The electrospray voltage was 2.0 kV. Peptides were analyzed by data-dependent MS/MS acquisition with a dynamic exclusion duration of 18 s. In MS1, the resolution was 70,000, the AGC target was 3e^6^, and the maximum injection time was 20 ms. In MS2, the resolution was 17,500, the AGC target was 1e^6^, and the maximum injection time was 60 ms. The scan range was 300 - 1400 m/z, and the 20 most intensive precursor ions were selected for MS/MS analysis.

### Data analysis

The raw data were processed using the Proteome Discoverer 1.3 proteomics platform. The fragmentation spectra were searched against the RefSeq Human database (20120320) using the Mascot search engine (v 2.2.06) with the precursor and fragment mass tolerances set to 10 ppm and 20 mmu, respectively. Two missed cleavage sites were allowed, and the minimum peptide length was 7 amino acids. Variable modifications: oxidation (M), and acetylation (protein N-terminal). Fixed modification: carbamidomethyl (C). Additionally, the deamidation of asparagine together with the incorporation of an ^18^O atom was set as a variable modification for the assignment of N-glycosites. Peptide ions were filtered using the cut-off scores of Percolator based on *p*-values < 0.01. The false discovery rate (FDR) was set to 1 % for peptide identifications. For label-free quantification, precursor ions areas were extracted using Proteome Discoverer 1.3 with a 10 ppm mass precision (the experimental m/z and retention times were recorded for precursor area quantification). Ratios for each peptide were normalized by the total identified peptides, and the ratios were used for N-glycopeptide-level quantification. 

### Bioinformatics and statistical analysis

To evaluate the trend of protein abundance changes, hierarchical clustering was performed and a distance tree was generated using Multi Experiment Viewer (MeV)[52]. To visualize the significantly regulated N-glycosites, Statistical Product and Service Solutions (SPSS) software was used. To study gene-disease relationships, the differentially regulated genes in metastatic cell lines were assigned to different diseases based on Disease Ontology and peer-reviewed evidence from GeneRIF using the web tool FunDO (http://django.nubic.northwestern.edu/fundo)[27]. Then, Cytoscape v.2.8.2 was used to visualize gene-disease interaction networks[53]. To explore the biological functions, subcellular localization and pathways and networks of the N-glycoproteins involved, Gene Ontology (GO) annotation[54], DAVID Bioinformatics Resources[29] and Ingenuity Pathways Analysis (IPA, Ingenuity Systems, Mountain View) were employed, respectively.

### Western-blotting analysis

The secretory proteins of metastatic HCC cell lines were resolved on 10 % SDS-PAGE gels, followed by transferring onto PVDF membranes (Millipore, USA). After incubation in blocking buffer (0.5 % Tween-20 in TBS, 5 % BSA) for 1 h at room temperature, membranes were blotted using antibodies against the targeted proteins for 1 h at room temperature. Membranes were then washed with TBST (TBS with 0.5% Tween-20) and incubated in 1:5,000 diluted HRP-conjugated IgG for 1 h at room temperature. After washing three times with TBST, the bands on the membrane were visualized using an ECL plus detection system[18]. 

## Supporting Information

Table S1 **The details of the protein identifications from the 8 replicate experiments, including the peak area information used for label-free quantitative analysis.**
(XLSX)

Table S2 **A total of 1,165 unique N-glycopeptides and 1,213 N-glycosites were recognized and mapped to 611 glycoproteins.**
(XLSX)

Table S3 **GO subcellular localization annotation of 611 N-glycoproteins identified.**
(XLSX)

Table S4 **The enrichment scores for the clusters of glycoproteins and secretory proteins were 195.2 and 50.2, respectively, as compared to the human proteome background by DAVID analysis.**
(XLSX)

Table S5 **The top 10 functional categories from IPA analysis.**
(XLSX)

Table S6 **The differentially expressed N-glycopeptides between MHCC97L and HCCLM3 cell lines.**
(XLSX)
